# Unconventional Vegetable Oils for a Reduction of Methanogenesis and Modulation of Ruminal Fermentation

**DOI:** 10.3389/fvets.2018.00201

**Published:** 2018-09-05

**Authors:** Danielle S. Freitas, Stephanie A. Terry, Rafael S. Ribeiro, Luiz G. R. Pereira, Thierry R. Tomich, Fernanda S. Machado, Mariana M. Campos, Patricia S. Corrêa, Adibe L. Abdalla, Rogério M. Maurício, Alexandre V. Chaves

**Affiliations:** ^1^Bioengineering Department, Federal University of São João del-Rei, São João del-Rei, Brazil; ^2^School of Life and Environment Sciences, Faculty of Science, University of Sydney, Sydney, NSW, Australia; ^3^Embrapa Dairy Cattle, Juiz de Fora, Brazil; ^4^Centre for Nuclear Energy in Agriculture, University of São Paulo, Piracicaba, Brazil

**Keywords:** exotic oils, sheep, *in vitro*, methane, cerrado biome

## Abstract

The objective of this study was to evaluate the use of vegetable oils from plants grown in Brazil, first using the *in vitro* batch culture, and then evaluating the oil with methane (CH_4_) reducing potential in an *in vivo* experiment. The *in vitro* experiment was conducted as a completely randomized design using the seven contrasting oils. Treatments consisted of a control and 3 increasing concentrations (0, 1, 2, and 5% v/v) of oil added to a tifton 85 hay samples. All vegetable oils linearly decreased (*P* < 0.01) gas production after 24 h of incubation, with the greatest reduction when 5% of oil was included into the diet. Açaí and buriti had no effect of CH_4_ (% or mL/g DM incubated) however carrot, macaúba, basil, passionflower, and pequi oil all linearly decreased (*P* < 0.01) CH_4_ production with increasing inclusion rate of oil. Pequi oil resulted in the largest decrease in CH_4_ production (mL/g DM incubated) after 24 h of *in vitro* incubation. The objective of the *in vivo* experiment was to evaluate the effects of pequi oil on nutrient digestibility, CH_4_ production, and rumen fermentation parameters in wethers fed a hay-based diet. The experiment was conducted as a 2 × 2 Latin Square design using 4 Dorper wethers (63.4 ± 1.46 kg body weight). There were 2 experimental periods of 21 d each, with d 1–14 used for diet adaptation and d 15–21 for measurements and collections. The treatments consisted of a control diet and pequi oil fed at 70 g per animal per day. The addition of pequi oil to the diet had no effect on feed intake or the digestibility of nutrients, however there was a numerical decrease in the population of cellulolytic bacteria. There was a tendency (*P* = 0.06) for pequi oil addition to decrease CH_4_ production (g/d) by 17.5%. From this study, we can conclude that pequi oil may be used as a suitable oil for reducing CH_4_ production from ruminants, with no negative effects on intake or digestibility.

## Introduction

Brazil has the fifth largest land mass and is home to the third largest cattle population. However due to increasing populations, it has been estimated that beef production in Brazil still needs to increase by 22.8% and dairy production by 29.8% to meet the demands for beef and milk products (1). Due to its large land mass, Brazil has high potential to meet these production needs (2). Increasing environmental awareness has also pressured agricultural sectors to seek alternative methods to reduce enteric methane (CH_4_) production from ruminants.

With restrictions placed on using great proportions of concentrates as well antibiotic use, there is a focus on “natural” products to manipulate rumen fermentation, particularly using plant-based products. Lipid supplementation is considered a promising strategy for dietary manipulation, with the capacity to decrease CH_4_ emissions without effecting performance (3). Lipids can act in different ways to suppress methanogenesis; including suppression of ciliate protozoa and archaea, biohydrogenation of free unsaturated fatty acids, reduction in organic matter fermentation, and replacing fermentable carbohydrates (4–6). However, the potential of individual lipid sources in reducing enteric CH_4_ production is dependent on the fatty acid composition (7–9).

Plant oil and oilseeds have been shown to consistently lower methane emissions over time without reduction in performance characteristics (10), however total fat content of the diet can only be increased up to 6–7% until feed intake is decreased (11). Several studies have compared the effects of supplementing different vegetable oils on feed intake and animal performance with results differing based on the type of oil supplemented (7–9).

The *Caryocar brasiliense* Camb tree, common name pequi, produces a fruit which is high in oil. This tree is endemic to Brazil, however is spread throughout South America (12). Pequi oil is high in the unsaturated fatty acid, oleic acid, as well as the saturated fatty acid palmitic acid. The oil has also shown to have antibacterial properties possibly due to high carotenoids concentration (13). Additionally, research facilities are currently interested in the cultivation of this tree, as well as its possible use as a feed resource (12). The objective of this study was to first investigate the effects of non-conventional oils derived from açaí, buriti, carrot, macaúba, basil, passionfruit, and pequi on rumen fermentation and CH_4_ production using the *in vitro* gas production technique and select the most promising oil for an *in vivo* study. Subsequently, the objective of the second study was to evaluate the effect of pequi oil on rumen fermentation, apparent nutrient digestibility, enteric CH_4_ production, and microbial population in sheep fed a hay-based diet.

## Materials and methods

The experiments were conducted at the Bioenergetic Laboratory of Multi-use Complex on Livestock Bio-efficiency and Sustainability of the Brazilian Agricultural Research Corporation, Embrapa (Coronel Pacheco, Minas Gerais, Brazil), Latitude: 21° 55′67′ 'S, Longitude: 43° 26′ 90″ W and altitude: 414 meters. The climate is classified as tropical with an average temperature of 25.6°C; relative humidity of 77% and annual mean rainfall of 1,400 mm.

All animal management and handling procedures were approved by Embrapa Dairy Cattle Animal Care and Use Committee. For the *in vitro* trial - Protocol CEUA—EGL n° 29/2015 and for the *in vivo*—Protocol CEUA No. 7080090616.

### *In vitro* study

#### Experimental design and treatments

The experiment was conducted as a completely randomized design with a control (100% Tifton 85 hay) and 3 increasing concentrations of vegetal oils [1, 2, and 5% (v/v)] sourced from Brazil (Table 1). The concentrations were chosen to obtain quadratic responses and calculated based on the volume of incubation media (e.g., 25 mL). Tifton 85 hay was used as the substrate and was ground to 1 mm and weighed into the incubation vials (0.5 g DM per vial) 1 d before incubation. The oils [açaí (*Euterpe oleracea*), buriti (*Mauritia flexuosa*), carrot (*Daucus carota*), macaúba (*Acrocomia aculeata*), basil (*Ocimum basilicum*), passionfruit (*Passiflora edulis*), and pequi (*Caryocar brasiliense*)] were obtained from Mundo dos Óleos (Brasilia, DF, Brazil), and were pipetted into each vial 1 h prior to incubation. The vegetable oils volumes added were 0.25, 0.5, and 1.25 mL for 1, 2, and 5% (v/v), respectively.

**Table 1:** Concentration of fatty acids (g/100 g of total fatty acids) identified in the oils used in *in vitro* batch cultures.

**Oils**	**C12:0**	**C14:0**	**C16:0**	**C18:0**	**C18:1*n*-9**	**C18:2*n*-6**	**C18:3*n*-3**	**C20:0**
Açaí	–	–	17.7	4.46	21.9	45.5	7.54	0.71
Buriti	0.37	0.43	18.1	3.91	38.0	29.4	6.61	1.43
Carrot	9.77	2.20	13.7	3.99	21.4	33.9	4.07	0.47
Macaúba	36.0	6.34	5.99	1.59	13.5	2.55	0.42	0.14
Basil	–	–	23.3	4.55	30.9	34.8	3.45	–
Passionfruit	0.32	0.22	15.51	3.98	22.5	44.9	3.34	1.01
Pequi	0.08	0.12	27.1	2.57	44.7	21.1	1.95	0.21

*C12:0, Lauric acid; C14:0, Myristic acid; C16:0, Palmitic acid; C18:0, Stearic acid; C18:1 n-9, Oleic acid; C18:2 n-6, Linoleic acid; C18:3 n-3, Linolenic acid; C20:0, Arachidic acid*.

### *In vitro* technique

Rumen inoculum was pooled from 3 ruminally cannulated crossbred non-lactating dairy cows (Holstein × Gyr), 2 h after feeding. Cows were fed a total mixed ration (TMR) consisting of corn silage, Tifton 85 hay, concentrate (forage to concentrate; 53:47% DM) and a minerals mixture. Rumen contents were filtered by gauze. The fluid was then placed in a thermos heated to 39°C and immediately taken to the laboratory.

The *in vitro* incubations were conducted using bottles (50 mL) sealed with rubber stoppers. On the day of incubation, bottles with the substrate were kept at 39°C in an incubator and continuously carbonated with CO_2_ at the time of incubation. To begin the incubation, 25 mL of culture medium was dispensed into each vial, then immediately sealed and placed into the incubator. Culture medium consisted of ruminal fluid and buffer solution at a ratio of 1:2, respectively (14). The incubator was maintained at 39 ± 0.5°C and bottles were kept on a rotary shaker at 90 oscillation/min.

#### Sample analysis

The gas volume was quantified at 8 and 24 h of incubation using water displacement technique (15) and the total production was calculated by adding the values obtained in the two readings. Gas samples were taken to quantify methane (CH_4_) production after 8 h of incubation, where 10 mL of gas were collected by inserting a 20 mL syringe through the septum, before gas pressure measurement. This gas was then transferred to previously evacuated 5.9 mL exetainers (Labco Ltd., High Wycombe, England). From each exetainer, 3 mL was removed for CH_4_ analysis (GC-FID Equipment Agilent Technologies 7820A-CH4: Gas Phase Chromatograph, Agilent Technologies, Model 7820A, Chromatography GC-FID EzChrom Elite interface software). After measuring the gas production at 24 h, the rubber stoppers were removed, and the pH was measured using a digital pH meter (Tecnopon, model MPa-210, Brazil) calibrated at 39°C. Immediately the vials were placed on ice to inhibit fermentation. Incubation media samples (1.5 mL) were taken after the 24 h of incubation and were placed in 0.3 mL of 20% metaphosphoric acid (w/v). Samples were kept at −20°C and then analyzed acetic, propionic, iso-butyric butyric acid, iso-valeric, and valeric acid using a high-performance liquid chromatography (HPLC; Waters, Model alliance separation module e2695 with a photodiode array detector; wavelength 210 nm; Waters Technologies of Brazil Ltd, Barueri, SP, Brazil) with a C18 ODS 80A (150 × 4.6 × 5 μm) column.

#### Determination of fatty acid profile of oils

For the analytical quantification of fatty acids, the oils were previously lyophilized, with samples containing between 10 and 50 mg of total fatty acids and subjected to direct transesterification and extraction by acid catalysis (16). For the analysis a gas-phase chromatograph (CG-ECD Agilent Technologies 6890N-SF6: ChemStation interface software with capillary column; HP-FFAP, 25 m × 0.2 mm × 0.33 μm). The stationary phase of acid was modified with nitroterephthalic polyethylene glycol using H_2_ as a carrier gas at 1.0 mL/min.

#### Statistical analysis

The data were analyzed using the mixed model procedure of SAS (SAS Inc., 2018, SAS OnlineDoc 9.1.4 Cary, NC, USA). The univariate procedure of SAS was used to test for normal distribution of data. The statistical design was completely randomized with 3 vials used per treatment, with 3 incubations runs. Fixed effects were oil, oil concentration, and the interaction between these terms. Random effects were incubation run and treatment within incubation run. Each incubation run (*n* = 3) was considered as the experimental unit. Treatment means for each level were compared using the least squares mean linear hypothesis test. Linear and quadratic effects were evaluated by using planned orthogonal polynomial coefficients for each parameter when Type 3 tests for fixed effects were ≤ 0.05. Significance was declared at *P* ≤ 0.05 and tendencies 0.05 < *P* ≤ 0.10.

### *In vivo* study

#### Animals, experimental design, and treatments

Due to its CH_4_ mitigation potential, *in vitro*, pequi oil was chosen to be fed in the metabolism trial. Four castrated Dorper wethers (63.4 ± 1.46 kg live weight) were used for the metabolism trial and for measurement of CH_4_ production using a respiration chamber designed to accommodate small ruminants. Animals were housed in metabolic crates throughout the experimental period. The experiment was conducted as a randomized 2 × 2 Latin square (2 × 2 periods, 2 animals per treatment). Animal were fed a total mixed ration (TMR) twice daily at 0730 h and 1530 h, *ad libitum* (5% orts), and allowed constant access to clean water (Table 2). The diets were formulated using the Small Ruminant Nutrition System (Version 1.9.6290.40564) to meet the nutritional requirements of wethers for maintenance. The diets were formulated to be iso-nitrogenous and iso-fibrous. Two diets were evaluated: control (without oil) and a diet with daily inclusion of pequi oil (Mundo dos Óleos) at 70 g oil per animal (75 mL oil/animal). The experiment periods consisted of a 14-d adaptation period, with 5 d of collection for digestibility parameters and 2 d of gas measurements using respiration chambers.

**Table 2:** Chemical composition and ingredients of the diet.

	**Treatment**	
	**Control**	**Pequi**
**INGREDIENTS, % DM**		
Tifton 85 hay	82.98	82.98
Corn grain	14.31	14.31
Mineral supplement^a^	2.72	2.72
Pequi oil (g/d)^b^	–	70
**CHEMICAL COMPOSITION, %DM (MEANS** ±**SD)**		
Dry matter (%)	83.2 ± 0.47	81.1 ± 0.45
Ash	7.8 ± 1.36	7.8 ± 1.30
Crude protein	8.8 ± 0.43	8.6 ± 0.41
Neutral detergent fiber (NDF)	69.9 ± 0.06	70.0 ± 0.06
NDF insoluble protein (NDFIP)	3.30 ± 0.02	3.29 ± 0.02
Ether extract (EE)^*c*^	1.4 ± 0.03	7.4 ± 0.03
Non-fiber carbohydrates (NFC)	12.1 ± 1.71	6.2 ± 1.63

*NFC, 100 – (CP + Ash + NDF + EE); SD, standard deviation*.

a *Ca 135 g/kg; P 50 g/kg' Na 195 g/kg; Zn 2000 mg/kg; Cu 190 mg/kg; F 500 mg/kg; Mn 1450 mg/kg, Co 20 mg/kg; I 20 mg/kg, Se 7 mg/kg*.

b *Pequi oil was fed at 70 g per animal per day*.

c *Estimated by the Small Ruminant Nutrition System (Version 1.9.6290.40564)*.

#### Apparent digestibility

For determination of apparent digestibility of nutrients, wethers were housed in metabolic crates (0.6 m width × 1.82 m length) during the study. The cage had a tray separating feces and urine, with total feces being collected and measured and collected each day for 5 days. Samples of feed, orts and feces were collected daily, pooled, and stored in plastic bags and kept at −20°C. Samples were pooled for each animal in each period, obtaining equal representative samples. For analysis, samples were dried at 55°C to constant weight and ground (1-mm sieve) using a Wiley mill.

#### Respiration chambers

During chamber measurements, sheep were housed in metabolism crate inside the respiration chamber for 2 periods of 22 h. All animals were previously adapted to stay in the chamber for the data collection phase and weighed before and after measurements. The open circuit respiration chamber (Intergado® Ltda., Contagem, MG, Brazil) had volume of 6.39 m^3^ (2.48 × 1.48 × 1.74 m) and made from aluminum and transparent polyethylene terephthalate glycol (PETG) walls to enhance the visual contact between the animals inside and outside the chamber. Air inside the chamber was maintained at 55% RH (relative humidity) and 22°C by an air conditioning unit (LG TSNH122H4W0, Manaus, Brazil) and a fan (Ventisol VM20-01, China) was used to circulate air within the chamber.

Inside the chamber, the fresh air inlet has a valve and a T connection fitted with 2 horizontal PVC tubes (50 mm diameter × 134 mm) punctured at each 10 cm with 1 cm wholes to avoid laminar flow, immediately above the feed bin and next to recirculating air entrance. The chamber was fitted with an air outlet which has a filter box (CSL-849-100HC, Solberg Manufacturing Inc., Itasca, IL), in the rear section of the ceiling, where the air was continuously drawn out through a 51-mm-diameter flexible polyurethane connected directly to a mass flowmeter (Flow Kit model FK-500, Sable International Systems, Las Vegas, NV). The flow rate used was 1.4 L/min for each kg of body weight (BW). The gas analysis and data acquisition system (Sable Systems International), as well the calibration procedures, were as previously described by Machado et al. (16), to calculate the CH_4_ production. The recovery for CH_4_ from the respiration system was 112.5 ± 5.23%.

Daily heat production (HP, Kcal/d) was calculated according to Brouwer (17): HP (Kcal/d) = (3,866 × O_2_) + (1,200 × CO_2_) – (0,518 × CH_4_).

#### Ruminal parameters

Rumen samples (~70 mL) were collected on d 19 of each period via the esophageal tubing. Rumen contents were squeezed through 4 layers of cheese cloth and pH was taken immediately (Model HI 8417, Hanna Instruments, Singapore). A 50 mL subsample was taken for DNA extraction and frozen at −20°C.

#### Ruminal microbial population—quantitative analysis

DNA extraction was performed using MOBIO PowerLyzer™ Power Soil (Geneworks, Australia). After extraction, DNA concentration and purity were checked using a ND-2000 spectrophotometer (Nanodrop Technologies). DNA samples were diluted to 10 ng/μL for standardization of concentration between samples. The quantification of the number of total bacterial genes, archaea*, Clostridium* sp., *Fibrobacter succinogenes*, anaerobic fungi*, Ruminococcus flavefaciens, Ruminococcus albus*, and *Selenomonas ruminantium* were estimated with specific primers (16S rRNA) by amplification of the ribosomal gene sequences and curves were generated for their quantification (Table 3). The efficiency of the amplification of each primer set between 84 and 100% and linear standard curve (R^2^) above 0.996 were accepted for subsequent calculation of gene copy number.

**Table 3:** Primers used for the quantitative analysis of ruminal microbial populations.

**Genera**	**Sequence of primers (5′a 3′)**	**Reference**
Total bacteria	F, CGGCAACGAGCGCAACCC R, CCATTGTAGCACGTGTGTAGCC	Denman and McSweeney (18); Denman et al. (19)
*Ruminococcus flavefaciens*	F, CGAACGGAGATAATTTGAGTTTACTTAGG R, CGGTCTCTGTATGTTATGAGGTATTACC	
*Fibrobacter succinogenes*	F, GTTCGGAATTACTGGGCGTAAA R, CGCCTGCCCCTGAACTATC	
Archaea	F, TTCGGTGGATCDCARAGRGC R, GBARGTCGWAWCCGTAGAATCC	
Protozoa	F, CTT CTT GCG AAC AGT TAG A R, CTC AAT GTC AAG CCC TGG	Asanuma et al. (20)
*Ruminococcus albus*	F, CGAACGGAGATAATTTGAGTTTACTTAGG R, CGGTCTCTGTATGTTATGAGGTATTACC	Yu et al. (21)
*Clostridium* sp.	F, GATAAGAGAGTGCTAGCTTGCTAGAA R, GTTAGCAACTAAATACGTGGGTTG	Yu et al. (21)
*Selenomonas ruminantium*	F, TGC GAA TAG TTT TTM GCA A R, CTC AAT GTC AAG CCC TGG	Asanuma et al. (20)
Fungi	F, CGAACGGAGATAATTTGAGTTTACTTAGG R, CGGTCTCTGTATGTTATGAGGTATTACC	Yu et al. (21)

The StepOnePlus™ (Applied Biosystems) thermocycler was used to quantify detection by real-time fluorescent PCR (Qpcr) with SYBR Green I as fluorescent dye. The reaction in a final volume of 10 μL containing 5 μL of the SYR Green ROX qPCR Master Mix (Thermo Scientific), 5 pmol of each primer (forward and reverse), 1 μL of DNA, and sterilized ultrapure water was applied. For the populations of *F. succinogenes, S. ruminantium, R. flavefaciens, R. albus*, and *Clostridium* sp., the relative quantification of the number of copies of the 16S rRNA gene from the ruminal microbiota groups was performed in relation to the number of total bacteria. For the populations of total bacteria, protozoa, fungi, and archaea the calculation was performed by absolute quantification using the standard curve method and expressed as the number of copies of amplicon from 16S rRNA that have been amplified by primers specific to each microorganism per 10 ng of DNA in 2 ml of ruminal fluid.

#### Chemical analysis

The following AOAC (22) methods, were applied to quantify dry matter (DM; Method 967.03), ash (Method 942), ether extract (EE) content by extraction with petroleum ether using a Soxtherm Fat Extractor (Gerhardt Instruments, Königswinter, Germany—Method 920.39). Neutral detergent fiber (NDF) concentration was quantified using of sodium sulfite (Na_2_SO_3_) (23) adapted for Ankom 200/220 Fiber Analyzer (Ankom Technol. Corp., Fairport, NY, EU) and not corrected for ash or protein. For this analysis, a thermostable alpha-amylase (Termamyl 2x) was added. The Kjeldahl method (Vapodest 20S, Gerhardt Instruments) with crude protein (CP) content calculated as N × 6.25 was applied for nitrogen (N) concentration determination. Non-fibrous carbohydrates (NFC, % of DM) was calculated using the equation NFC = 100–[CP + NDF + EE + Ash].

#### Statistical analysis

The data were analyzed as a 2 × 2 Latin square (2 treatments × 2 periods, 2 animals per treatment), using the SAS mixed model (SAS Inc., 2018, SAS OnlineDoc 9.1.4 Cary, NC, USA). The means were compared using the LSMEANS/DIFF, considering period and treatment as fixed effect and animal located within each treatment as random effect. The method for computing denominator degrees of freedom was Kenwardroger (e.g., DDFM = KENWARDROGER) Significance was declared at *P* ≤ 0.05 and tendencies 0.05 < *P* ≤ 0.10.

## Results

### *In vitro* study

There was an oil × concentration effect on gas production after 24 h incubation (mL or mL/g DM incubated; *P* = 0.02) for all oils, and a linear effect of concentration (*P* < 0.01) was observed (Table 4). Carrot, passionfruit and pequi had the lowest total gas production (ml/g DM incubated) after 24 h of incubation when oil was included at 5% of incubation media. There was an oil × concentration effect observed for CH_4_ data (% or mL/g DM incubated). Carrot, macaúba, basil, passionfruit, and pequi oils linearly decreased (*P* < 0.01) the % CH_4_ in the gas sample. When calculated based on mL/g DM incubated, buriti also decreased CH_4_ production. Pequi oil resulted in the largest reduction of CH_4_ production.

**Table 4:** Effect of different oils on accumulative gas and methane production in an *in vitro* batch culture using a hay-based diet.

**Oil**	**Conc (%, v/v)**	**Gas 24h (mL)**	**Gas 24h (mL/g DM incubated)**	**CH_4_ (%)**	**CH_4_ 24h (mL/g DM incubated)**
Açaí	0	67.5^ab^	133.5^ab^	11.1	15.1
	1	77.4^a^	152.8^a^	10.4	16.1
	2	71.2^ab^	140.7^ab^	11.5	16.3
	5	53.5^b^	105.5^b^	11.9	12.6
Buriti	0	67.5^a^	133.5^a^	11.1	15.1^a^
	1	76.7^a^	151.3^a^	12.2	18.3^a^
	2	74.6^a^	147.0^a^	12.0	17.4^a^
	5	41.2^b^	81.2^b^	9.2	7.7^b^
Carrot	0	67.5^a^	133.5^a^	11.1^a^	15.1^a^
	1	43.2^b^	85.4^b^	7.8^b^	6.9^b^
	2	26.7^bc^	52.7^bc^	5.7^b^	3.3^b^
	5	21.5^c^	42.6^c^	5.0^b^	2.5^b^
Macaúba	0	67.5^a^	133.5^a^	11.1^a^	15.1^a^
	1	48.1^b^	95.4^b^	8.0^ab^	7.7^b^
	2	43.6^b^	85.9^b^	8.3^a^	7.2^b^
	5	39.2^b^	77.8^b^	7.7^b^	6.1^b^
Basil	0	67.5^a^	133.5^a^	11.1^a^	15.1^a^
	1	58.6^ab^	116.0^ab^	7.8^b^	9.3^b^
	2	47.4^bc^	93.7^bc^	8.0^a^	7.6^b^
	5	36.1^c^	71.4^c^	7.0^b^	5.0^b^
Passionfruit	0	67.5^a^	133.5^a^	11.1^a^	15.1^a^
	1	49.6^ab^	97.8^ab^	8.0^ab^	7.9^b^
	2	35.1^bc^	72.0^bc^	5.2^bc^	4.2^bc^
	5	18.1^c^	35.8^c^	3.4^c^	1.3^c^
Pequi	0	67.5^a^	133.5^a^	11.1^a^	15.1^a^
	1	37.2^b^	73.7^b^	5.1^b^	3.9^b^
	2	28.7^bc^	56.8^bc^	5.6^b^	3.5^b^
	5	17.1^c^	34.0^c^	4.9^b^	2.0^b^
SEM		7.01	13.85	1.29	2.12
*P*-value	Oil	<0.01	<0.01	<0.01	<0.01
	Conc	<0.01	<0.01	<0.01	<0.01
	Oil × Conc	0.02	0.02	0.04	<0.01
	L	<0.01	<0.01	<0.01	<0.01
	Q	0.03	0.03	<0.01	<0.01

*Superscripts that differ within each column are significantly different within each oil treatment*.

*SEM, standard error of the means; Conc., concentration; L, linear; Q, quadratic*.

There was an oil × concentration effect (*P* ≤ 0.05) on pH, propionate, butyrate, BCVFA percentages of total VFA and the acetate to propionate (A:P) ratio (Table 5). The pH was linearly increased (*P* < 0.01) by increasing concentrations of oils included in the diet for all oils. The was a quadratic response (*P* < 0.01) of propionate percentage of total VFA to increasing concentration of oil in the diet for açaí, carrot, and pequi oil where propionate percentage of total VFA was increased at 1 and 2 % inclusion of oil. There was a linear and quadratic effect (*P* < 0.01) of buriti, carrot, passionfruit, basil, and pequi oils on acetate percentage of total VFA, where for most of oils butyrate percentage of total VFA was decreased at greater oil inclusion. All oils resulted in a quadratic response (*P* < 0.01) to BCVFA with increasing inclusion of oil. There was effect of açaí and pequi oil on the A:P, in which the ratio was decreased from the control at 1% oil inclusion and increased at 5% inclusion.

**Table 5:** Effect of different oils on pH and the volatile fatty acids (VFA) concentrations in an *in vitro* batch culture using a hay-based diet.

**Oil**	**Conc. (%, v/v)**	**pH**	**Total VFA, mmol/L**	**Acetate (A), mmol/100 mmol**	**Propionate (P), mmol/100 mmol**	**Butyrate, mmol/100 mmol**	**Valerate, mmol/100 mmol**	**BCVFA, mmol/100 mmol**	**A:P**
Açaí	0	6.19^ab^	97.1	64.7	15.7^a^	15.0	2.0	2.9^ab^	4.1^b^
	1	6.11^b^	102.5	63.3	17.6^a^	15.5	2.0	2.3^b^	3.6^b^
	2	6.18^ab^	82.3	64.5	15.0^a^	15.3	2.0	2.7^ab^	4.3^b^
	5	6.34^a^	63.9	67.5	11.5^b^	15.3	1.6	3.3^a^	5.9^a^
Buriti	0	6.19^ab^	97.1	64.7	15.7	15.0^a^	2.0	2.9^a^	4.1
	1	6.11^b^	89.3	65.1	14.5	15.4^a^	1.9	2.6^ab^	4.5
	2	6.09^b^	87.1	64.2	15.9	15.5^a^	2.0	2.2^b^	4.0
	5	6.35^a^	58.7	66.1	16.1	13.1^b^	1.8	2.0^b^	4.1
Carrot	0	6.19^b^	97.1	64.7	15.7^b^	15.0^a^	2.0	2.9^a^	4.1
	1	6.19^b^	78.3	64.3	20.1^a^	11.9^b^	1.9	1.2^b^	3.2
	2	6.45^a^	59.2	66.5	19.1^a^	9.9^b^	1.9	1.3^b^	3.5
	5	6.53^a^	57.9	68.0	17.3^ab^	10.3^b^	1.6	1.8^b^	3.9
Macaúba	0	6.19^b^	97.1	64.7	15.7	15.0^a^	2.0	2.9^a^	4.1
	1	6.28^ab^	65.8	66.5	16.4	12.7^b^	1.8	2.0^b^	4.1
	2	6.39^a^	71.7	64.8	16.2	13.5^ab^	2.2	2.9^a^	4.0
	5	6.42^a^	61.9	64.8	16.8	14.1^ab^	2.0	2.9^a^	3.9
Manjericão	0	6.19^b^	97.1	64.7	15.7	15.0^a^	2.0	2.9^a^	4.1
	1	6.24^ab^	78.8	64.6	17.2	13.8^ab^	2.1	2.2^b^	3.8
	2	6.37^ab^	73.5	64.3	17.3	13.6^ab^	2.0	2.8^ab^	3.7
	5	6.43^a^	61.8	65.3	15.7	12.3^b^	1.6	2.9^ab^	4.2
Passionfruit	0	6.19^b^	97.1	64.7	15.7	15.0^a^	2.0	2.9^a^	4.1
	1	6.06^b^	76.7	65.4	18.0	12.0^b^	1.8	2.4^ab^	3.6
	2	6.52^a^	54.1	68.1	17.6	9.6^c^	1.7	2.0^b^	3.9
	5	6.66^a^	44.5	69.9	16.0	9.5^c^	1.4	2.0^b^	4.4
Pequi	0	6.19^b^	97.1	64.7	15.7^b^	15.0^a^	2.0	2.9^a^	4.1^b^
	1	6.43^a^	65.0	65.3	22.0^a^	9.5^b^	1.8	1.2^b^	3.0^b^
	2	6.46^a^	52.3	65.7	21.8^a^	10.5^b^	1.8	1.5^b^	3.0^b^
	5	6.48^a^	44.9	69.0	15.8^b^	10.2^b^	1.6	2.4^a^	4.4^a^
SEM		0.08	10.08	1.48	1.17	0.80	0.12	0.27	0.30
*P*-value	Oil	<0.01	0.03	0.14	<0.01	<0.01	0.07	<0.01	<0.01
	Conc.	<0.01	<0.01	<0.01	<0.01	<0.01	<0.01	<0.01	<0.01
	Oil × Conc.	0.05	0.64	0.62	<0.01	<0.01	0.46	<0.01	0.03
	L	<0.01	<0.01	<0.01	0.07	<0.01	<0.01	0.28	<0.01
	Q	0.26	0.22	0.56	<0.01	<0.01	0.01	<0.01	<0.01

*Superscripts that differ within each column are significantly different within each oil treatment*.

*SEM, standard error of the means; Conc., concentration; L, linear; Q, quadratic; BCVFA, branched-chain volatile fatty acids (iso-butyric + iso-valeric)*.

### *In vivo* study

Pequi oil addition had no effect (*P* ≥ 0.46) on the apparent digestibility of nutrients (Table 6). Feed intake was not affected by pequi oil (*P* ≥ 0.27; Table 7). There was a tendency (*P* = 0.06) for an effect of pequi oil on CH_4_ production (g/d) in which CH_4_ was reduced by 17.5%. There was also a tendency for decreased CH_4_ yield based on g/kg DMI (*P* = 0.10) and g/kg NDF intake (*P* = 0.10). There was no effect of pequi oil on heat production (*P* = 0.12).

**Table 6:** Effect of pequi oil on apparent digestibility of nutrients in wethers fed a hay-based diet.

	**Treatment**		**SEM**	***P*-value**
	**Control**	**Pequi**		
**APPARENT DIGESTIBILITY, %**				
Dry matter	62.0	60.8	2.12	0.59
Organic matter	63.5	62.0	1.93	0.46
Crude protein	52.2	53.0	2.66	0.77
Neutral detergent fiber	54.5	52.7	2.48	0.49

*SEM, standard error of the means*.

**Table 7:** Effect of pequi oil on dry matter intake (DMI), methane production and heat production in wethers fed a hay-based diet.

	**Treatments**		**SEM**	***P*-value**
	**Control**	**Pequi**		
Live body weight (kg)	63.7	63.1	2.07	0.77
Dry matter intake (g/d)	1168.1	1181.1	10.54	0.27
Dry matter intake (g/kg BW)	51.9	52.8	1.54	0.61
CH_4_ (g/d)	31.5	26.0	2.38	0.06
CH_4_ (g/kg DMI)	29.9	24.6	2.65	0.10
CH_4_ (g/kg NDF intake)	49.6	42.6	3.56	0.10
Heat production (Kcal/kg BW)	104.6	115.7	6.14	0.12

*SEM, standard error of the means; BW, body weight; NDF, neutral detergent fiber*.

Pequi oil had no effect (*P* ≥ 0.52) on the concentration of total bacteria, archaea, protozoa or fungi (Table 8). Except for *R. albus* (0.79), the relative expression in terms of fold-changes of specific groups of the rumen microbiota after supplementation of pequi oil showed reduction ranging from −0.037 to −0.882 (Table 8).

**Table 8:** Effect of pequi oil on rumen microbial diversity^a^ in wethers fed a hay-based diet fold-changes for specific groups after supplementation of pequi oil.

	**Treatment**		**SEM**	***P*-value**	
	**Control**	**Pequi**			**Fold-changes^b^**
Total bacteria	19.05	19.07	0.465	0.98	−0.037
*Ruminococcus flavefaciens*	8.47	7.81	0.662	0.52	−0.212
*Fibrobacter succinogenes*	10.19	8.27	1.177	0.31	−0.882
*Ruminococcus albus*	7.87	9.22	1.757	0.62	0.791
*Clostridium* sp.	14.45	13.48	86.82	0.99	−0.300
Archaea	7.42	6.74	0.718	0.54	−0.401
Protozoa	7.04	6.45	0.678	0.56	−0.301
Fungi	8.75	7.68	1.07	0.52	−0.245

a *Copies of amplicon from 16S rRNA that have been amplified by primers specific to each microorganism. Data have been natural log (LN) transformed*.

b *Fold-changes for specific groups after supplementation of pequi oil. Log2 (Pequi/Control): Vargas et al. (24). SEM, standard error of the means*.

## Discussion

Oil supplementation can exert suppressive effects on digestibility and VFA production due to inhibitory effects on rumen microbiota. In this study, total gas production was decreased with increasing concentration of oils added to the substrate. It is well established that high concentrations of oil to the diet can inhibit fermentation (23, 25) therefore the maximum fat inclusion in the diet is recommended at no greater than 6% (26). However, in this study, all vegetable oils decreased total gas production when included at only 5% v/v. The reduction in gas production can explain the increase in pH with increasing concentration of oil supplementation, as high concentrations of oils increase pH, due to inhibition of fermentation (27).

All oils decreased CH_4_ production (%), except for açai and buriti, on a linear scale. Pequi oil had the greatest suppressive effect on CH_4_ (mL/g DM incubated), decreasing production after 24 h incubation by 86.1% and for this reason it was selected to use in the animal trial. Methane reduction potential of fats has been associated with the concentrations of MUFA and PUFA (28) with C12:0 and C18:3 having a high inhibitory effects for methanogenesis. Pequi oil may have been the most effective in decreasing CH_4_ due to its high palmitic (C16:0) and oleic acid (C18:1) content, probably acting in cellulolytic bacteria and archaea, respectively (29).

However, buriti also has a high concentration of these fatty acid and only suppressed CH_4_ production (mL/g DM incubated) when included at 5%. Additionally, buriti also has a high concentration of the PUFA, alpha-linolenic acid [C18:3 (n-3)] and linoleic acid [C18:2 (n-6)] compared to the other examined oils, however, the same potential for CH_4_ reduction was not exhibited. The passionfruit and carrot oil were also notable in decreasing CH_4_ (%) inducing 55.0%, and 69.4% reductions. Of these CH_4_ suppressing oils, only carrot and pequi oil changed the VFA profile. Both carrot and pequi oil increased the percentage of propionate, however only pequi oil supplementation altered the acetate to propionate ratio.

Whilst gas production was reduced *in vitro*, dry matter intake and other digestibility parameters were not influenced in the metabolism component of the study. However, pequi oil did have a tendency to decrease CH_4_ production by 17.5% when fed at 70 g per animal per day.

In contrast to this, Duarte et al. (30, 31) demonstrated that pequi oil included at 1.5 mL/d, increasing the dietary fat content to 6.0%, had no effect on *in vitro* CH_4_ production using the rumen simulation technique (RUSITEC). However, authors state that this may have been due to the small concentration of pequi oil tested in relation to fermentation liquid. Polyphenolic compounds (e.g., tannins) originating from tropical leaves or shrubs negatively impact methanogenic production by direct reduction of archaea population (32). However, during extraction of the oil from pequi pulp polyphenolic compounds were also identified (33). Therefore, the lower methane emission observed in lambs fed pequi oil in this work could be a result of the associative effects of certain fatty acids and polyphenolic compounds present in the oil that could contribute to the change in microbial populations and consequently decrease enteric CH_4_ production.

Lipids can also act to suppress the protozoal populations. However, in the *in vivo* study, protozoa communities were not affected by pequi oil supplementation. Comparatively, in the *in vitro* study butyrate production was decreased with increasing supplementation with pequi oil. Butyrate production is associated with certain microbial populations, specifically protozoa (34, 35). A decrease in protozoa numbers are associated with a decrease in CH_4_ production as the main fermentation end products of protozoal activity are acetate and butyrate, two VFA which provide H_2_ for methanogenesis (36).

The lack of change of microbial diversity is supported by Duarte et al. (31) who found that the archaeal and bacterial microbiota structure was not affected by pequi oil supplementation using the rumen simulation technique. However they did report that pequi oil increased the relative abundance of *Anaerovibrio*, a genus that has reported to be involved in the metabolism of lipids in the rumen, enhancing propionate synthesis (37, 38). In our study, when we compared the changes in microbial communities due to changes in the diet, i.e., addition of pequi, within the same animal (*n* = 2), we observed that except for *Ruminococcus albus*, all other studied cellulolytic bacteria were reduced, as observed with the fungi, archaea, and protozoa populations (39, 40).

## Conclusion

From the *in vitro* study it can be concluded that all the vegetable oils decreased gas production after 24 h incubation, however only carrot, macaúba, basil, passionfruit, and pequi oil decreased CH_4_ (% and mL/g DM incubated). Pequi oil had the greatest CH_4_ reducing potential and also decreased the acetate to propionate ratio.

Supplementing pequi oil into a hay-based diet of wethers had no effect on the apparent digestibility of nutrients, dry matter intake or microbial diversity. Pequi oil supplementation did however, numerically decrease CH_4_ production by 17.5%. Therefore, pequi oil may be daily fed at 70 g/animal to reduce enteric CH_4_ production without any negative effects on feed digestibility.

## Author contributions

AC and LP study design. DF and RR conducting *in vitro* study. DF, RR, TT, and FM conducting *in vivo* study. DF, AA, LP, RM, and RR lab analysis. AA DNA extraction and PCR analysis. AC, ST, and AA statistical analysis. ST, DF, AC, RM, and LP writing the manuscript. LP, FM, MC, and RM leader of the grants. AC, ST, LP, RM, TT, AA, FM, and MC editing the final version of the manuscript. All authors read and approved the final manuscript.

### Conflict of interest statement

The authors declare that the research was conducted in the absence of any commercial or financial relationships that could be construed as a potential conflict of interest.
